# A deep transcriptomic analysis of pod development in the vanilla orchid (*Vanilla planifolia*)

**DOI:** 10.1186/1471-2164-15-964

**Published:** 2014-11-07

**Authors:** Xiaolan Rao, Nick Krom, Yuhong Tang, Thomas Widiez, Daphna Havkin-Frenkel, Faith C Belanger, Richard A Dixon, Fang Chen

**Affiliations:** Department of Biological Sciences, University of North Texas, 1155 Union Circle #305220, Denton, TX 76203 USA; Samuel Roberts Noble Foundation, 2510 Sam Noble Parkway, Ardmore, OK 73402 USA; Unité Reproduction et Développement des Plantes, INRA (UMR879)/CNRS (UMR5667)/Université de Lyon and Ecole Normale Supérieure de Lyon, 69364 Lyon, France; Department of Plant Biology and Pathology, Rutgers, The State University of New Jersey, 59 Dudley Road, New Brunswick, NJ 08901 USA

**Keywords:** Vanilla, *Vanilla planifolia*, Transcriptome, RNA sequencing, Lignin, Vanillin

## Abstract

**Background:**

Pods of the vanilla orchid (*Vanilla planifolia*) accumulate large amounts of the flavor compound vanillin (3-methoxy, 4-hydroxy-benzaldehyde) as a glucoside during the later stages of their development. At earlier stages, the developing seeds within the pod synthesize a novel lignin polymer, catechyl (C) lignin, in their coats. Genomic resources for determining the biosynthetic routes to these compounds and other flavor components in *V. planifolia* are currently limited.

**Results:**

Using next-generation sequencing technologies, we have generated very large gene sequence datasets from vanilla pods at different times of development, and representing different tissue types, including the seeds, hairs, placental and mesocarp tissues. This developmental series was chosen as being the most informative for interrogation of pathways of vanillin and C-lignin biosynthesis in the pod and seed, respectively. The combined 454/Illumina RNA-seq platforms provide both deep sequence coverage and high quality de novo transcriptome assembly for this non-model crop species.

**Conclusions:**

The annotated sequence data provide a foundation for understanding multiple aspects of the biochemistry and development of the vanilla bean, as exemplified by the identification of candidate genes involved in lignin biosynthesis. Our transcriptome data indicate that C-lignin formation in the seed coat involves coordinate expression of monolignol biosynthetic genes with the exception of those encoding the caffeoyl coenzyme A 3-*O*-methyltransferase for conversion of caffeoyl to feruloyl moieties. This database provides a general resource for further studies on this important flavor species.

**Electronic supplementary material:**

The online version of this article (doi:10.1186/1471-2164-15-964) contains supplementary material, which is available to authorized users.

## Background

Vanilla is the most popular flavoring material in the United State [1]. Of the approximately 110 Vanilla species (Orchidaceae, monocot) that have been identified, only two are important in terms of commerce and cultivation; *Vanilla planifolia* Andrews and *V. tahitensis* JW Moore [2]. Among these, *V. planifolia* is the most valued for its flavor qualities and is therefore widely cultivated and used for the production of food additives [2, 3]. The fully-grown mature fruits of vanilla, also called beans or pods, develop characteristic aromatic properties by the process of curing. The cured beans are referred to as vanilla [2], and the major flavor compound is vanillin (3-methoxy, 4-hydroxy-benzaldehyde).

In spite of its economic importance, there is little genetic diversity and few genetic or genomic resources in *V. planifolia.* The flavor industry generally disfavors genetically modified crops, so there have been virtually no attempts to modify the quantity or quality of the flavor of the vanilla bean through biotechnological approaches. Nevertheless, a better understanding of the genetic complement of *V. planifolia* could provide information on the still-disputed biosynthetic route to vanillin [4], support mechanistic studies on other areas of the novel biochemistry of this species, such as the biosynthesis of novel seed coat lignins [5], and provide molecular markers to advance non-transgenic breeding programs targeting flavor quality, yield or disease resistance.

The chromosome number for *V. planifolia* has been reported as 2n = 32. Most *V. planifolia* accessions are considered to be diploid with a 2C-value of 5.03 pg [6], but due to the large size and complexity of the *V. planifolia* genome, limited sequence resources are currently available.

The rapid development of next-generation sequencing (NGS) technologies has enabled the efficient and economical high-throughput sequencing of entire genomes or transcriptomes [7, 8]. NGS systems are typically represented by Roche’s 454 GS FLX and Illumina/Solexa Genome Analyzer. In general, Roche’s sequencing technology produces long reads and is advantageous for assembly of sequences into longer contigs; however, the number of reads generated in each run is lower than that of other platforms and not enough to reach deep coverage for low-abundance genes. The Illumina technology provides a high number of reads for deeper coverage, which is beneficial for gene discovery [9, 10]. Although its short read length limits de novo contig assembly efficiency [9, 10], various strategies, algorithms and software have been developed for short-read assembly, especially for de novo assembly in the absence of a reference genome [11].

Using the above technologies, we have generated very large gene sequence datasets from vanilla pods at different times of development, and representing different tissue types, including the seeds, hairs, and placental and mesocarp tissues within the pod. Based on previous studies [5, 12, 13], this developmental series was chosen as reflecting the times of synthesis and accumulation of vanillin and catechyl lignin in the pod and seed, respectively. The annotated sequence data provide a foundation for understanding multiple aspects of the biochemistry and development of the vanilla bean, as exemplified by the identification of candidate genes involved in lignin biosynthesis and interrogation of their expression patterns in the developing pod and seed in relation to the accumulation of the C-lignin polymer in the seed coat [4].

## Results and discussion

### Biochemistry of greenhouse-grown vanilla beans

To determine if the green vanilla beans obtained from greenhouse-grown material retain the biosynthetic capabilities of plants grown in nature, we measured the vanillin content of the greenhouse grown vanilla bean. Without acid hydrolysis, only a very low amount of vanillin could be detected in the green beans. After acid hydrolysis, 0.53 g of vanillin per100g fresh green bean was measured in the six-month old vanilla beans, indicating that the beans used in this study maintain the biosynthetic capability of naturally-grown beans as regards glucovanillin accumulation. The ability of greenhouse-grown plants to accumulate C-lignin in the seed coats has been reported previously [5].

### Next generation sequencing and assembly of reads

**Figure 1:**
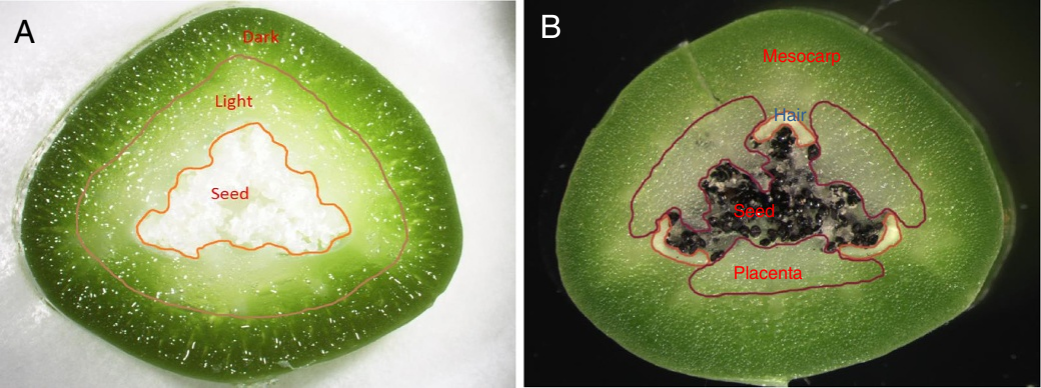
**Vanilla pod tissue samples for RNA isolation. (A)** A six-week old vanilla pod cross section. The pods at earlier developmental stages were separated into three tissue samples (dark, light and seed). **(B)** A twelve-week old vanilla pod cross section. The pods at later developmental stages were separated into four tissue samples (mesocarp, placenta, hair cells, and seed).

To obtain an overview of *V. planifolia* gene expression profiles in different organs at different developmental stages, RNA was extracted and cDNA prepared from 26 different tissue samples. The tissue types within the pod are shown in Figure 1, and the listing of all tissue samples given in Additional file 1. cDNA derived from RNA from the two biological replicates of each of the above tissue samples was sequenced by the Illumina platform, and a single pool of cDNA obtained by mixing all cDNA samples was sequenced using the Roche 454 platform. For the Illumina sequences, purity filtered reads ranged from 0.69 to 1.8 million reads per sample and the percentage of preserved reads ranged from 82.4% to 97.4%. Pooling of the Roche 454 sequencing runs resulted in 1,678,293 qualified reads with an average length of 397 bases after filtering and trimming (Additional file 2).

To obtain transcript expression information in each tissue or organ, each of 26 vanilla Illumina libraries was assembled separately using a combination of the programs Velvet [14] and Oases [15]. Velvet currently takes short reads, removes errors, and then produces high quality unique contigs. Oases uploads a preliminary assembly produced by Velvet, and then clusters the contigs into small groups, called loci. It then exploits the paired-end read and long read information, when available, to construct transcript isoforms [14, 15]. Using these approaches, transcripts per library ranged from 44,403 to 94,637 with mean transcript length ranging from 1,694 to 2,159 bp (Additional file 3). To demonstrate the quality of assembled transcripts, the size distribution of four selected samples is shown in Additional file 4.

**Figure 2:**
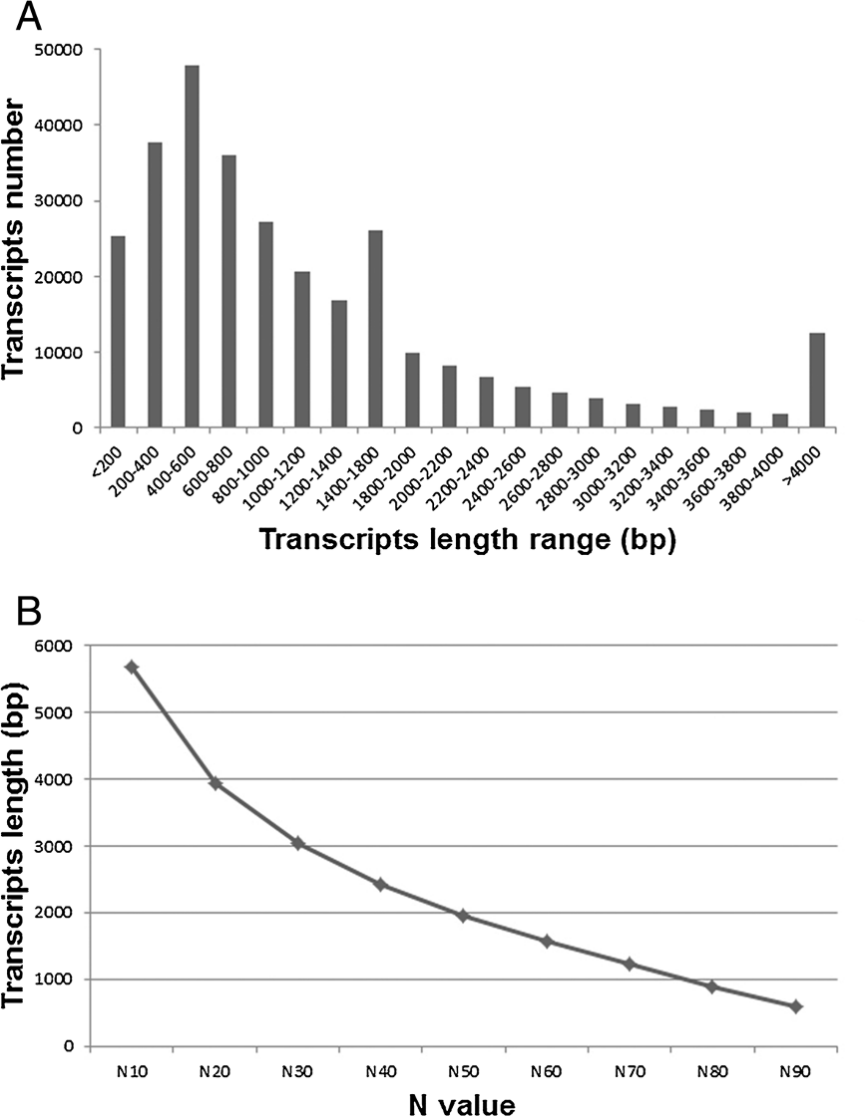
**Length distribution of the hybrid-assembled transcripts. (A)** Length frequency distribution of the 301,459 transcripts, **(B)** Size distribution plot of the 301,459 transcripts.

An often used combination of current sequencing technologies is to mix de-novo 454 assembly and Illumina mapping assemblies; the 454 approach allows the building of long contigs, and the Illumina approach reduces problematic 454-generated homopolymer sequences [16]. Therefore, to produce high quality vanilla transcripts, we employed an optimized two-step strategy. First, a combined assembly of all 26 Illumina samples (3,439,193,362 reads total) was produced using Velvet 1.2.03 with 27 k-mer length, producing 6,371,646 contig sequences. Then, Illumina contigs and qualified 454 reads were assembled together with MIRA 3.2.1 [16, 17]. This resulted in a total of 301,459 contigs which were considered as vanilla-expressed transcripts for further annotation and analysis. The size distribution of the total 301,459 contigs is shown in Figure 2. The contig N50 is the length of the smallest contig in the set that contains the largest contigs whose combined length represents at least 50% of the contig assembly; this parameter is generally used as a standard metric for assembly size [18]. Here size distribution at N50 was 1960 bp in length and average contig size was 1256 bp. All short reads obtained in this study have been submitted to the NCBI Sequence Read Archive (SRA) [BioProject: PRJNA253813]. Accession numbers for each library are listed at “Availability of supporting data” section. All assembled data can be searched and retrieved at http://www.sc.noble.org/vanilla/blast/blast.php.

For quality control, we mapped our results to another recently produced *V. planifolia* sequence dataset that did not include multiple tissue types. This consisted of 372 M bases of short 454 reads generated by the University of Copenhagen and deposited at [NCBI: SRX286672]. The combined assembled transcripts were mapped using Blast, and the results showed excellent consistency between the 454 reads in the public domain and the vanilla transcripts generated in this project. A total of 96.6% of the 454 reads from [NCBI: SRX286672] were able to be mapped back to 82,595 combined assembled transcripts. For further quality control analysis, we randomly selected 35 contigs and designed primers for RT-PCR amplification. In this analysis, 32 out of the 35 primer sets successfully resulted in a band of the expected size (Additional file 5), and the identities of all the resulting cloned sequences were confirmed by Sanger sequencing.

### Mapping of transcripts to reference databases

**Figure 3:**
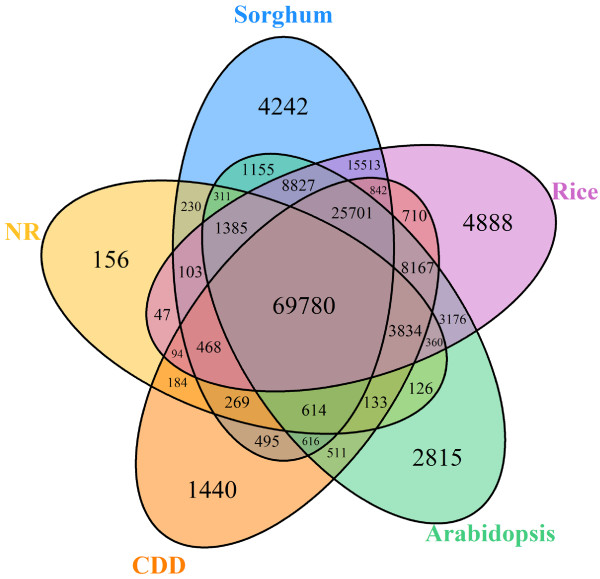
**Venn diagram showing the number of annotated V. planifolia genes from five different databases.** The numbers of annotated transcripts found among the non-redundant (NR) NCBI database, the Conserved Domains Database (CDD), and the Arabidopsis, rice, and sorghum proteomes are shown.

The 301,459 vanilla transcripts were compared to five reference databases (the non-redundant (NR) NCBI database; the Conserved Domains Database (CDD); and the Arabidopsis, rice, and sorghum proteomes) using BlastX with a cut-off E-value of 1E-5. Using this approach, over 52% of all vanilla transcripts (157,191 transcripts) returned an above cut-off BLAST result in at least one of these five reference databases (Additional file 6). The homology search against NR resulted in 130,550 annotated genes. Seventy eight thousand and ninety three genes were identified as encoding proteins with conserved domains, while 113,857 genes, 127,510 genes and 143894 genes were identified as encoding proteins homologous to proteins in the Arabidopsis, rice and sorghum proteomes, respectively. The overlap of these five database annotations is shown in the Venn diagram in Figure 3.

### An integrated digital gene expression database

To estimate transcript expression levels in each of the 26 Illumina libraries, the reads were mapped back to the assembled transcript sequences using Bowtie2 2.0.0 [19]. The number of reads mapped to each transcript were then counted and used to calculate the reads per kilobase of transcript per million reads mapped (RPKM) for every transcript in each library (Additional files 7, 8 and 9).

In the integrated gene expression database, the expression levels of a total of 301,459 transcripts are presented in 26 different tissues or organs. Most of these genes (183,724) showed above 1 RPKM value in at least one tissue or organ. Furthermore, 37,538 transcripts showed above 10 RPKM value in at least one tissue or organ, and 2,156 transcripts were above 100 RPKM value in at least one tissue or organ.

**Figure 4:**
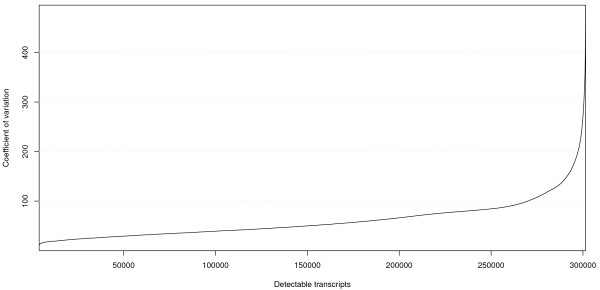
**Distribution of coefficient of variation (CV) for all detectable genes in the vanilla expressed gene dataset.** The CVs of digital expression level among 26 organ and tissue samples were sorted in ascending order and plotted for each transcript using R software.

The coefficient of variance (CV) reflects the variance of gene expression levels in each library. The coefficient of variance ranged from 0 to 496% for expressed genes, with an average of 60% (Figure 4). To identify a set of constitutively expressed genes, we applied several filters, including a CV of <40%, transcript level of >100 in at least one library, and a transcript ratio <4 when comparing the highest transcript level in any library with the lowest level in any library for a given gene. A set of 154 genes was identified as encoding transcripts that changed little during development, and 131 genes in the set were annotated with functions. Amongst these genes are homologs of common housekeeping genes, such as genes for ubiquitin (Ubi), actin and actin-like protein. Other potential reference genes include genes encoding glucose-6-phosphate dehydrogenase (G6PDH), HSP90 and ubiquitin-conjugating enzymes. These stably expressed genes, listed in Additional file 10, can serve as reference genes for normalizing transcript levels of other vanilla genes prior to comparative gene expression analysis, as commonly used in gene expression atlas studies in other species [20, 21].

For further validation of reference genes in Vanilla, two frequently used housekeeping genes, Actin1 and ubiquitin (Ubi), were selected from the stably expressed genes list, and their expression levels were evaluated in early stage seeds and in stem by quantitative real-time RT-PCR. Actin1 showed a similar constitutive expression pattern to that of Ubi, but with higher expression level (Additional file 11). The data are consistent with our digital gene expression database, and Actin1 was chosen as the reference gene for further gene expression analyses. Another group also recently identified Actin and EF1 to be the most stably expressed reference genes during pod development in V. *planifolia* [22].

**Figure 5:**
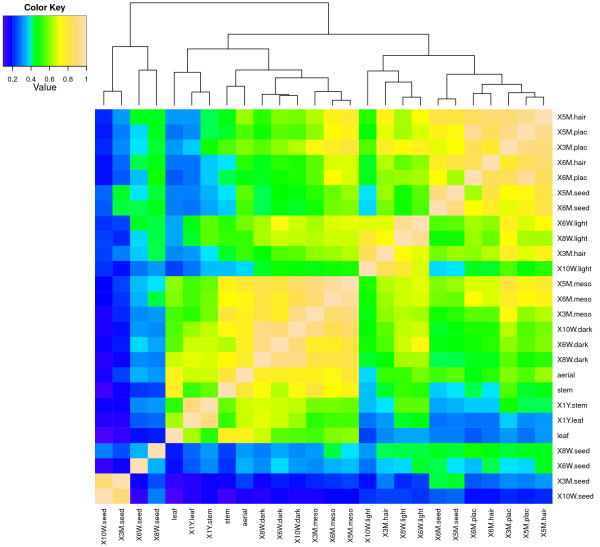
**Correlation matrix of 26 vanilla RNA-seq libraries.** Pairwise Pearson correlation coefficients (PCC) were calculated for comparison among transcriptomes of various vanilla tissues and organs. Samples were hierarchically clustered with the Euclidean distance method. The color scale indicates the degree of correlation. The correlation matrix and heat map were generated using R software.

Similarity in transcript profiles was assessed by Pearson correlation and cluster (PCC) analysis among tissue samples. Overall, PCC values ranged from 0.099 (leaves vs. 10-week seed) to 0.96 (5-month mesophyll vs. 6-month mesophyll). As expected, transcript profiles in seed tissues from 6 weeks to 3 months were poorly correlated with transcript profiles in other tissues (PCC <0.50) (Figure 5). Lignification in the seed begins after 8 weeks [5]. Among seed tissues, the transcript profiles in early developmental stages (6 and 8 weeks) showed low correlation to each other, while a high correlation was observed in seed transcript expression profiles at 10 weeks and 3 months. This indicates that there are many changes in gene expression occurring during early seed development, but little after the seed matures.

### Annotation of tissue-specifically expressed genes

**Figure 6:**
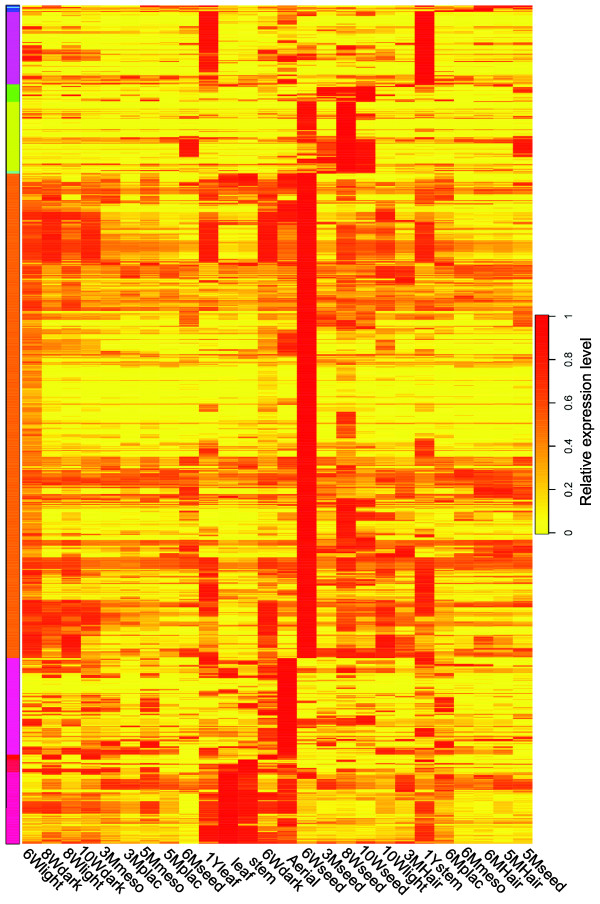
**Tissue-specific gene expression heatmap.** Transcripts with RPKM values at least three times as high in the indicated tissue than in any other tissues were selected and clustered using R with the package ‘rsgcc’. The color scale indicates the tissue specificity score associated with all transcripts preferentially expressed in the various tissues and organs. The tissue specificity score is 1, if the gene is only expressed in one tissue.

Knowledge of genes that are expressed specifically in one organ or tissue, or genes that are expressed at substantially higher levels in some organs/tissues than in others can provide insights into specialized processes at work in these organs/tissues [20]. To detect genes with tissue-restricted expression, all transcripts were filtered to obtain those above 10 RPKM value in at least one tissue or organ, then further analysed in R with the package “rsgcc” [23], setting the parameter value as “0.75”, which means that the expression level of a gene must be at least 3 times higher in one tissue than in any other tissue. We found 590 genes that were exclusively expressed in 6 week seeds, and 83 genes exclusively expressed in 8 week seeds. Eighty seven genes were more highly expressed in leaf than in any other organ; 119 genes were more highly expressed in aerial roots; and 93 genes were more highly expressed in the 1 year old stem (Additional file 12). These data are summarized in the heatmap in Figure 6.

After sorting the tissue-specifically expressed genes into Gene Ontology (GO) categories using Plant GOslim ancestor terms (http://www.geneontology.org/) [24] and the KEGG pathway database (http://www.genome.jp/kegg/) [25, 26], we found that genes involved in primary metabolic processes, biosynthesis processes and cellular metabolic processes were highly expressed in 6 week old seed (Additional file 13A). The 6 week seed specific gene set was rich in genes annotated as having involvement in starch and sucrose metabolism pathways in the KEGG database (Additional file 13, under KEGG enzyme code EC:2.4.1.34, EC:2.4.1.29, EC:3.2.1.26, EC:2.4.1.12 and EC:3.2.1.48). Genes with transport, trans-membrane transport and response to stimulus functions in the GO categories were found to be more highly expressed in the aerial root-specific gene group (Additional file 13B). In the leaf, most genes were annotated as encoding photosystem I and II proteins.

There were only a few specific marker genes for the major organ systems. More tissue-specific markers were found for roots and seeds than for leaves, perhaps reflecting the more specialized nature of these organ systems [27]. These subsets of organ-specific genes will be a useful tool for research into biological processes during Vanilla pod development.

### Identification of genes potentially involved in lignin and vanillin biosynthesis

**Figure 7:**
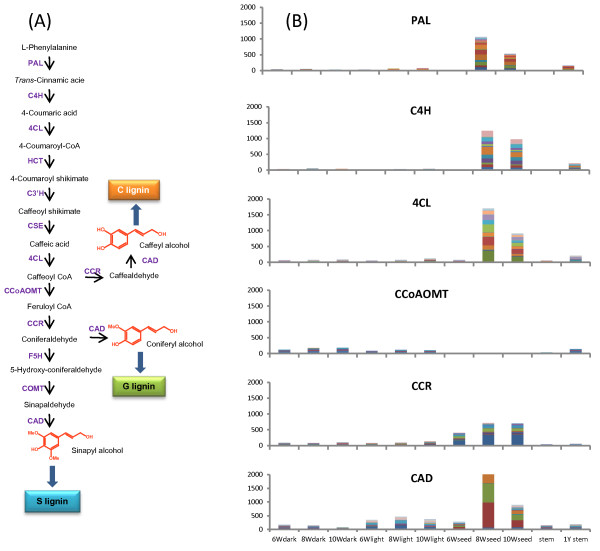
**Lignin biosynthesis pathway and lignin-related gene expression in the early stage seeds and stems. (A)** A simplified lignin biosynthesis pathway. **(B)** The expression levels of PAL, 4CL, CCR, CAD, C4H and CCoAOMT were represented as RPKM values in the light and dark pod tissues, seeds at 6, 8 and 10 weeks, and in stems. Each color represents one contig annotated as the corresponding class of lignin-related gene.

Lignin is a phenylpropanoid polymer commonly found in terrestrial plant secondary cell walls. Lignin is assembled by oxidative polymerization of three major monolignols, *p*-hydroxycinnamyl alcohol (H unit), coniferyl alcohol (guaiacyl or G unit) and sinapyl alcohol (S unit). However, during the early stages of seed development in *V. planifolia*, a newly discovered polymer (C-lignin) is deposited to high concentrations in the seed coat. This lignin is naturally synthesized from the unusual monolignol caffeyl alcohol, whereas lignin in the vegetative tissues of *V. planifolia* is of the normal guaiacyl/syringyl (G/S) type [5]. To address potential pathways of C-lignin biosynthesis, we used annotated lignin pathway genes in Arabidopsis as query sequences (Figure 7A), searched by BlastX with the threshold as E-value <1E-5, and searched against the NCBI NR, GO and KEGG databases with the annotation of lignin pathway. Finally, 233 Vanilla genes were identified as homologous to genes potentially involved in the monolignol pathway (Additional file 14). These included genes encoding phenylalanine ammonia-lyase (PAL; 35 contigs), coumaroyl shikimate 3′-hydroxylase (C3′H; 5 contigs), cinnamate 4-hydroxylase (C4H; 14 contigs), 4-coumarate: CoA ligase (4CL; 41 contigs), cinnamoyl CoA reductase (CCR; 26 contigs), hydroxycinnamoyl CoA:shikimate hydroxycinnamoyl transferase (HCT; 9 contigs), caffeoyl-CoA 3-*O*-methyltransferase (CCoAOMT; 7 contigs), caffeic acid 3-*O*-methyltransferase (COMT; 1 contigs; characterized in [28, 29]), and caffeoyl-CoA *O*-methyltransferase-like (named as OMT-4 and OMT-5; 8 contigs; characterized in [30]), cinnamyl alcohol dehydrogenase (CAD; 23 contigs), caffeoyl shikimate esterase (CSE; 64 contigs) [31].

**Figure 8:**
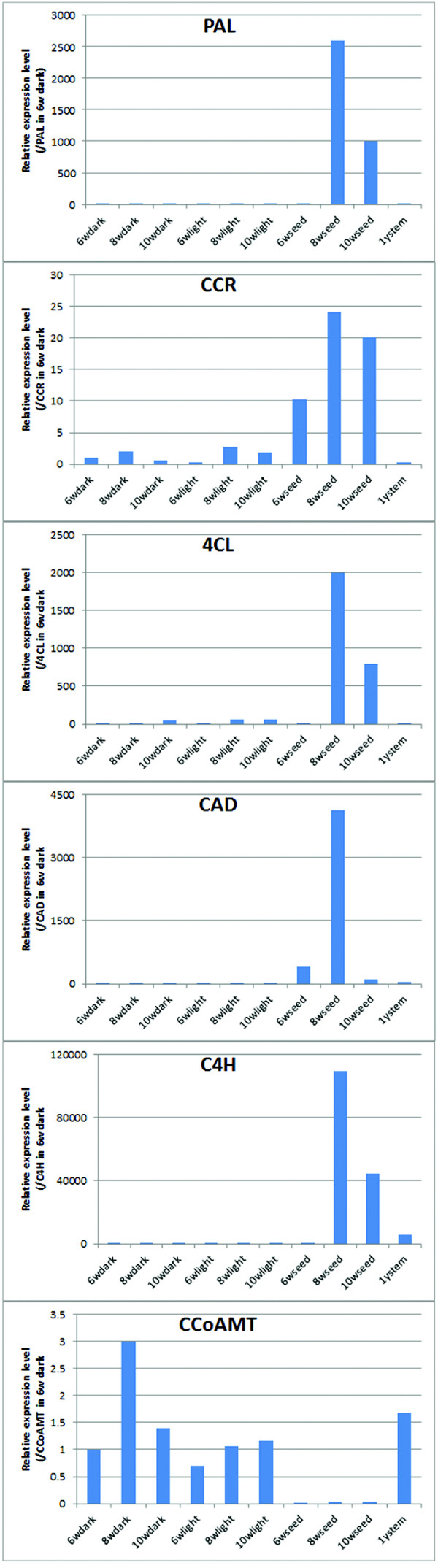
**Validation of lignin-related gene expression in the early stage seeds and stems.** qRT-PCR was performed to verify the expression patterns of PAL, 4CL, CCR, CAD, C4H and CCoAOMT in the light and dark pod tissues, seeds at 6, 8 and 10 weeks and in stems. Expression values were normalized to expression levels of actin, and the expression value in 6 W dark was set to 1. The results are the means of duplicate measurements of two biological replicates, and p-values for difference in expression between 8 week seed tissue and 6 week dark pod tissues were less than 0.01 (Student’s t-test) for all transcripts tested.

Most of the predicted lignin-related genes shared similar expression patterns in pod tissues, with highest expression in seeds at 10 weeks post-pollination, and with lowest expression in the dark and light mesocarp tissues (Figure 7B). This pattern was observed for most of the contigs representing each gene. However, although CAD transcripts were, overall, found at highest level in developing seeds, some CAD contigs were more highly expressed in the light mesocarp tissue. More strikingly, CCoAOMT transcripts were virtually absent from developing seeds, in spite of the high expression level of the other lignin biosynthetic genes. These digital expression patterns were confirmed by quantitative real-time RT-PCR analysis of selected contigs corresponding to PAL, 4CL, CCR, CAD, C4H and CCoAOMT in early developmental stages of seeds as well as in stems. The sequences of the primers used for RT-PCR are given in Additional file 15. Consistent with the digital expression database, PAL, 4CL, CCR, CAD and C4H displayed very high transcript levels in 8 week seeds and relatively high levels in 10 week seeds, while low transcript levels in dark and light mesocarp tissues. In contrast, CCoAOMT transcripts were detected in dark and light mesocarp tissues, but barely in seed tissues (Figure 8). The low level of CCoAOMT transcripts is likely the biochemical basis for the production of the novel non-methylated C-lignin in the vanilla seed coats [5]. This result is consistent with our earlier finding of low OMT enzyme activities in the C-lignin containing seed coat of *Cleome hassleriana* [32]. It is currently not clear how the low expression of the *O*-methyltransferases is regulated in vanilla seeds. Deep mining of transcription factors in the transcriptome database should help us to answer this question.

The digital expression database contains transcript level values for all potential lignin pathway genes in additional pod tissues such as hairs and placenta at later stages of pod development. This represents the timing and location of the accumulation of glucovanillin [33]. The vanillin pathway likely shares several reactions with the monolignol pathway [29]; genes specifically dedicated to vanillin biosynthesis remain to be described, although forms of PAL [22] and *O*-methyltransferase [4] have been suggested be involved in the process. The present work will facilitate the documentation of genes important for the formation of glucovanillin and other flavor compounds, and such results will be reported elsewhere.

## Conclusions

Next-generation sequencing and computational data mining tools have been used for high-throughput transcriptomic profiling of developing vanilla beans. The combined 454/Illumina RNA-seq platforms provide both deep sequence coverage and high quality de novo transcriptome assembly for this non-model crop species. The annotated sequence data provide a foundation for understanding multiple aspects of the biochemistry and development of the vanilla bean, as exemplified by the identification of candidate genes involved in an unusual lignin biosynthesis pathway in vanilla seed. Our transcriptome data indicate that C-lignin formation in the seed coat involves coordinate expression of monolignol biosynthetic genes with the exception of those encoding the caffeoyl coenzyme A 3-*O*-methyltransferase for conversion of caffeoyl to feruloyl moieties. This database provides a general resource for further studies on this important flavor species.

## Methods

### Plant materials and RNA isolation

Vanilla (*Vanilla planifolia*) vines were grown in the greenhouse in MetroMix 350 soil mix at 20–23°C (24–28°C in summer) with 45-60% relative humidity with a 16 h day from 6:00 h to 22:00 h, 150 μmol/m^2^/s light intensity, facilitated by supplementary lighting. Plants were watered daily with weekly addition of Scotts peat-lite 20-10-20 fertilizer at 100 ppm N. The flowers were hand pollinated in March and April of 2011. Green vanilla beans were harvested periodically after pollination. The selected 26 different tissue samples varied from early stages of pod development (seed, placental laminae (“light” for short) and mesocarp (“dark” for short) tissues at 6 , 8 and 10 weeks post-pollination), mature pods (seed, V-shaped hair cells (“hair” for short), placental laminae, and mesocarp tissues at 3, 5 and 6 months post-pollination), leaf and stem from mature plants harvested at 6 months and 1 year post-pollination of the flowers, and aerial roots harvested from one year old stems. Before tissue separation, the beans were cut into 2 cm sections to facilitate the isolation of seeds from mesocarp and placental tissue. At the later stages (after 3 months), the hair cells were also isolated from the placentae cells. The isolated vanilla pod tissues and seeds were immediately frozen in liquid nitrogen and ground to a fine powder with a mortar and pestle, and then stored at −80°C until RNA isolation using TRI Reagent (Molecular Research Center, Inc. Cincinnati, OH) according to the manufacturer’s protocol. Briefly, about 100 mg of tissues were mixed with 1 ml of TRI reagent in 2.0 ml Eppendorf tubes, and the samples allowed to stand for 10 min at room temperature. Chloroform (0.2 ml) was added, and the tubes were capped and shaken vigorously by vortexing followed by centrifugation at 12,000 × g for 10 min at 4°C. The upper aqueous phases containing RNA were transferred to fresh tubes and 0.5 ml of isopropanol was added and mixed. The samples were allowed to stand for 10 min at room temperature and were then centrifuged at 12,000 × g for 10 min at 4°C to precipitate the RNA. The supernatant was removed and the RNA pellet washed with 1 ml of 75% ethanol and centrifuged at 10,000 × g for 5 min. The RNA was briefly dried under air and re-dissolved in water.

### Determination of vanillin content in greenhouse-grown vanilla bean

Vanillin content of mature vanilla beans obtained from the greenhouse was measured using HPLC after acid hydrolysis. Briefly, the six month old green vanilla beans were ground to a fine powder under liquid nitrogen. The samples (three replicates) were then extracted with 70% methanol for 4 h at room temperature. After removing the solid residue by centrifugation, the methanolic solutions were dried under nitrogen gas and hydrolysed with 20% (V/V) HCl at 100°C for 1 h and subject directly to HPLC. The HPLC system consisted of a Thermo UltiMate 3000 HPLC system (Thermo Scientific, Sunnyvale, CA) and a Phenomenax C 18 column (250 × 4.6 mm 5 μ) (Phenomenex Inc, Torrance, CA). The mobile phase used was A: 1% phosphoric acid, B: acetonitrile. Gradient: 8% B for 5 min, 8% B to 35% B over 45 min, then 100% for 10 min. Commercial vanillin from Sigma-Aldrich (St. Louis, MO) was used as a standard for identification and construction of a standard curve.

### RNAseq library construction and next-generation sequencing

For each sample, 1 μg of total RNA was used for RNA-seq library construction using TruSeq RNA Sample Prep Kits v2 (Illumina Inc., San Diego, CA) according to the manufacturer’s instructions at the Genomics Core Facility at the Noble Foundation. Each library was indexed. Six libraries with different indexes each were pooled together to run on one Hiseq2000 lane targeting 100 bp paired reads. The Hiseq2000 run was conducted at the Genomics Core Facility of the Oklahoma Medical Research Foundation, Oklahoma City.

### De novo transcript assembly and construction of a digital gene expression atlas

Processing of the 100 bp paired-end Illumina reads began by interleaving the read mates for each sample into a single file and trimming bases with quality scores of 20 or less from the end of each read. Reads less than 40 bp long after trimming were discarded along with their mates.

Each of the 26 vanilla Illumina libraries was assembled separately using a combination of Velvet [14] and Oases [15]. Velvet was run using a k-mer length of 55 and an average insert length of 300 bp. Expected coverage and coverage cut-off levels were set to be automatically determined by the program. The results of the Velvet assemblies were then run through Oases using an insert length of 300 bp, a minimum transcript length of 200 bp, a coverage cut-off of 5, and an edge fraction cut-off of 0.25.

A combined assembly of all 26 Illumina samples (3,439,193,362 reads total) was produced using Velvet 1.2.03 with a k-mer length of 27, producing 6,371,646 contig sequences. A library of 1,678,293 454 reads (mean length =397.5 bp) was also produced and quality trimmed using a cut-off score of 20. The 454 reads and Illumina contigs were then assembled together with MIRA 3.2.1 [17], using the “normal” quality parameters for a de novo 454 EST assembly.

To estimate transcript expression levels in each of the 26 Illumina libraries, the reads were mapped back to the assembled transcript sequences using Bowtie2 2.0.0 (beta 7) [19] with the “sensitive” end-to-end alignment parameters. The number of reads mapped to each transcript was then counted and used to calculate the RPKM value for every transcript in each library.

### Sequence analysis and gene annotation

The resulting 301,459 transcripts from de novo combined assembly were annotated via alignment with the NCBI NR database, the Conserved Domains Database, and the Arabidopsis, rice, and sorghum proteomes (ftp://ftp.ncbi.nlm.nih.gov/). Tissue specific genes were detected in R 3.0.1 [34] with the package “rsgcc” [23]. Tissue specific genes were further annotated with default parameter values using Blast2Go [35]. After the Blast2Go mapping process, EC numbers from the KEGG pathway (http://www.genome.jp/kegg/) and GO terms were generated.

### Real-time quantitative RT-PCR

Total RNA was isolated from seed, mesocarp and stem tissues as described above. cDNA synthesis was performed using DNase I-treated RNAs and Superscript III reverse transcriptase (Invitrogen). Primer pairs for real-time qRT-PCR were designed using GenScript Real-time PCR Primer Design software (https://www.genscript.com/ssl-bin/app/primer) with the default parameters (Additional file 15). qRT-PCR was performed in duplicate for each sample and at least two biological replicates were evaluated for each gene tested. The qRT-PCR reactions were performed in an optical 96-well plate with a PikoReal.

Real-Time PCR System (Thermo Scientific), using SYBR Green Master Mix reagent (Simga) with PCR program as previously described [36]. Data were collected and analysed using PikoReal Software (Thermo Scientific). PCR efficiency was estimated using PikoReal software [37] and transcript levels were determined by relative quantification [38] using the actin gene as a reference.

## Availability of supporting data

The data sets supporting the results of this article are available in the NCBI Sequence Read Archive (SRA) repository, NCBI SRA accession No.: SRX627422, SRX634866, SRX634867, SRX634872, SRX634873, SRX634874, SRX634907, SRX634908, SRX634909, SRX648194, SRX648209, SRX648428, SRX648438, SRX648439, SRX648555, SRX648556, SRX648557, SRX648564, SRX648565, SRX648567, SRX648568, SRX648569, SRX648570, SRX648571, SRX648572, SRX648573, SRX648574.

## Electronic supplementary material

Additional file 1: **Tissue sample list.** (XLSX 10 KB)

Additional file 2: **Statistics of the raw and trimmed RNA-seq data.** (XLSX 13 KB)

Additional file 3: **Statistics of the assembly transcripts for each tissue or organ.** (XLSX 11 KB)

Additional file 4: **Length frequency distribution of the assembly transcripts from Seed, Hair, Meso and Plac in 3 month, respectively.** (PDF 1 MB)

Additional file 5: **PCR validation for the hybrid-assembled transcripts.** (PDF 1 MB)

Additional file 6: **Function annotation for all hybrid-assembled transcripts.** (XLSX 19 MB)

Additional file 7: **RPKM values for all hybrid-assembled transcripts in 26 tissues or organs.** (XLSX 18 MB)

Additional file 8: **RPKM values for all hybrid-assembled transcripts in 26 tissues or organs (continue).** (XLSX 18 MB)

Additional file 9: **RPKM values for all hybrid-assembled transcripts in 26 tissues or organs (continue).** (XLSX 3 MB)

Additional file 10: **Most stably expressed genes in the development of vanilla.** (XLSX 46 KB)

Additional file 11: **Q-PCR validation for Ubi and Actin.** **(A)** Cq values of Ubi and Actin detected by Q-PCR in 6 weeks, 8 weeks, and 10 weeks of dark, light and seed tissues and in stems, **(B)** Correlation of Cq values of Ubi and Action. (PDF 843 KB)

Additional file 12: **Function annotation for tissue-specific genes.** (XLSX 108 KB)

Additional file 13: **GO classification of tissue-specific genes in 6 week seeds and aerial roots.** (PDF 2 MB)

Additional file 14: **Lignin-related genes list with RPKM values.** (XLSX 49 KB)

Additional file 15: **Primers for PCR and QPCR.** (XLSX 13 KB)

## References

[1] Correll D (1953). Vanilla-its botany, history, cultivation and economic import. Econ Bot.

[2] Ramachandra Rao S, Ravishankar GA (2000). Vanilla flavour: production by conventional and biotechnological routes. J Sci Food Agric.

[3] Simmonds NW (1982). Review of J. W. Purseglove, E. G. Brown, C. L. Green, and S. R. J. Robbins ‘Spices’. Exp Agric.

[4] Dixon RA, Havkin-Frenkel D, Belanger FC (2011). Vanillin Biosynthesis- not as Simple as it Seems?. Handbook of Vanilla Science and Technology.

[5] Chen F, Tobimatsu Y, Havkin-Frenkel D, Dixon RA, Ralph J (2012). A polymer of caffeyl alcohol in plant seeds. Proc Natl Acad Sci U S A.

[6] Bory S, Catrice O, Brown S, Leitch IJ, Gigant R, Chiroleu F, Grisoni M, Duval MF, Besse P (2008). Natural polyploidy in *Vanilla planifolia* (Orchidaceae). Genome.

[7] Metzker ML (2010). Applications of next-generation sequencing technologies - the next generation. Nat Rev Gene.

[8] Wall PK, Leebens-Mack J, Chanderbali AS, Barakat A, Wolcott E, Liang H, Landherr L, Tomsho LP, Hu Y, Carlson JE, Ma H, Schuster SC, Soltis DE, Soltis PS, Altman N, dePamphilis CW (2009). Comparison of next generation sequencing technologies for transcriptome characterization. BMC Genomics.

[9] Su CL, Chao YT, Alex Chang YC, Chen WC, Chen CY, Lee AY, Hwa KT, Shih MC (2011). De novo assembly of expressed transcripts and global analysis of the *Phalaenopsis aphrodite* transcriptome. Plant Cell Physiol.

[10] Liu L, Li Y, Li S, Hu N, He Y, Pong R, Lin D, Lu L, Law M (2012). Comparison of next-generation sequencing systems. J Biomed Biotechnol.

[11] Garber M, Grabherr MG, Guttman M, Trapnell C (2011). Computational methods for transcriptome annotation and quantification using RNA-seq. Nat Methods.

[12] Joel DM, French JC, Graft N, Kourteva G, Dixon RA, Havkin-Frenkel D (2003). A hairy tissue produces vanillin. Israel J Plant Sci.

[13] Odoux E, Brillouet J-M (2009). Anatomy, histochemistry and biochemistry ofr glucovanillin, oleoresin and mucilage accumualtion sites in green mature vanilla pod (*Vanilla planifolia*; Orchidaceae): a comprehensive and critical reexamination. Fruits.

[14] Zerbino DR, Birney E (2008). Velvet: algorithms for de novo short read assembly using de Bruijn graphs. Genome Res.

[15] Schulz MH, Zerbino DR, Vingron M, Birney E (2012). Oases: robust de novo RNA-seq assembly across the dynamic range of expression levels. Bioinformatics.

[16] Chevreux B, Pfisterer T, Drescher B, Driesel AJ, Müller WEG, Wetter T, Suhai S (2004). Using the miraEST assembler for reliable and automated mRNA transcript assembly and SNP detection in sequenced ESTs. Genome Res.

[17] Chevreux B, Wetter T, Suhai S (1999). Genome sequence assembly using trace signals and additional sequence information. Comput. Sci. Biol.: Proc. German Conference on Bioinformatics GCB'99.

[18] Miller JR, Koren S, Sutton G (2010). Assembly algorithms for next-generation sequencing data. Genomics.

[19] Langmead B, Trapnell C, Pop M, Salzberg SL (2009). Ultrafast and memory-efficient alignment of short DNA sequences to the human genome. Genome Biol.

[20] Zhang JY, Lee YC, Torres-Jerez I, Wang M, Yin Y, Chou WC, He J, Shen H, Srivastava AC, Pennacchio C, Lindquist E, Grimwood J, Schmutz J, Xu Y, Sharma M, Sharma R, Bartley LE, Ronald PC, Saha MC, Dixon RA, Tang Y, Udvardi MK (2013). Development of an integrated transcript sequence database and a gene expression atlas for gene discovery and analysis in switchgrass (*Panicum virgatum* L.). Plant J.

[21] Benedito VA, Torres-Jerez I, Murray JD, Andriankaja A, Allen S, Kakar K, Wandrey M, Verdier J, Zuber H, Ott T, Moreau S, Niebel A, Frickey T, Weiller G, He J, Dai X, Zhao PX, Tang Y, Udvardi MK (2008). A gene expression atlas of the model legume Medicago truncatula. Plant J.

[22] Fock-Bastide I, Palama TL, Bory S, Lecolier A, Noirot M, Joet T (2014). Expression profiles of key phenylpropanoid genes during *Vanilla planifolia* pod development reveal a positive correlation between *PAL* gene expression and vanillin biosynthesis. Plant Physiol Biochem.

[23] Ma C, Wang X, Ma MC (2013). rsgcc: Gini Methodology-Based Correlation and Clustering Analysis of Microarray and RNA-Seq Gene Expression Data. R package version 1.0.6 edn.

[24] Ashburner M, Ball CA, Blake JA, Botstein D, Butler H, Cherry JM, Davis AP, Dolinski K, Dwight SS, Eppig JT, Harris MA, Hill DP, Issel-Tarver L, Kasarskis A, Lewis S, Matese JC, Richardson JE, Ringwald M, Rubin GM, Sherlock G (2000). Gene ontology: tool for the unification of biology. The Gene Ontology Consortium. Nat Genet.

[25] Kanehisa M, Goto S (2000). KEGG: Kyoto encyclopedia of genes and genomes. Nucleic Acids Res.

[26] Kanehisa M, Goto S, Sato Y, Kawashima M, Furumichi M, Tanabe M (2014). Data, information, knowledge and principle: back to metabolism in KEGG. Nucleic Acids Res.

[27] Schmid M, Davison TS, Henz SR, Pape UJ, Demar M, Vingron M, Scholkopf B, Weigel D, Lohmann JU (2005). A gene expression map of *Arabidopsis thaliana* development. Nat Genet.

[28] Li HM, Rotter D, Hartman TG, Pak FE, Havkin-Frenkel D, Belanger FC (2006). Evolution of novel *O*-methyltransferases from the *Vanilla planifolia* caffeic acid *O*-methyltransferase. Plant Mol Biol.

[29] Pak FE, Gropper S, Dai WD, Havkin-Frenkel D, Belanger FC (2004). Characterization of a multifunctional methyltransferase from the orchid *Vanilla planifolia*. Plant Cell Rep.

[30] Widiez T, Hartman TG, Dudai N, Yan Q, Lawton M, Havkin-Frenkel D, Belanger FC (2011). Functional characterization of two new members of the caffeoyl CoA *O*-methyltransferase-like gene family from *Vanilla planifolia* reveals a new class of plastid-localized *O*-methyltransferases. Plant Mol Biol.

[31] Vanholme R, Cesarino I, Rataj K, Xiao YG, Sundin L, Goeminne G, Kim H, Cross J, Morreel K, Araujo P, Welsh L, Haustraete J, McClellan C, Vanholme B, Ralph J, Simpson GG, Halpin C, Boerjan W (2013). Caffeoyl shikimate esterase (CSE) is an enzyme in the lignin biosynthetic pathway in Arabidopsis. Science.

[32] Tobimatsu Y, Chen F, Nakashima J, Escamilla-Trevino LL, Jackson L, Dixon RA, Ralph J (2013). Coexistence but independent biosynthesis of catechyl and guaiacyl/syringyl lignin polymers in seed coats. Plant Cell.

[33] Havkin-Frenkel D, French JC, Graft NM, Pak F, Frenkel C, Joel D (2004). Interrelation of curing and botany in vanilla (*Vanilla planifolia*) bean. Acta Hort.

[34] R Core Team (2013). R: A Language and Environment for Statistical Computing.

[35] Conesa A, Gotz S, Garcia-Gomez JM, Terol J, Talon M, Robles M (2005). Blast2GO: a universal tool for annotation, visualization and analysis in functional genomics research. Bioinformatics.

[36] Czechowski T, Stitt M, Altmann T, Udvardi MK, Scheible WR (2005). Genome-wide identification and testing of superior reference genes for transcript normalization in Arabidopsis. Plant Physiol.

[37] Ramakers C, Ruijter JM, Deprez RH, Moorman AF (2003). Assumption-free analysis of quantitative real-time polymerase chain reaction (PCR) data. Neurosci Lett.

[38] Pfaffl MW (2001). A new mathematical model for relative quantification in real-time RT-PCR. Nucleic Acids Res.

