# Supplementary figures and images for: A deep transcriptomic analysis of pod development in the vanilla orchid (Vanilla planifolia)
Source: BMC Genomics. 2014 Nov 7;15(1):964. doi: 10.1186/1471-2164-15-964 (PMC4233054; doi:10.1186/1471-2164-15-964)

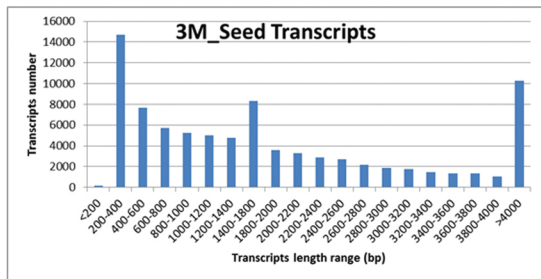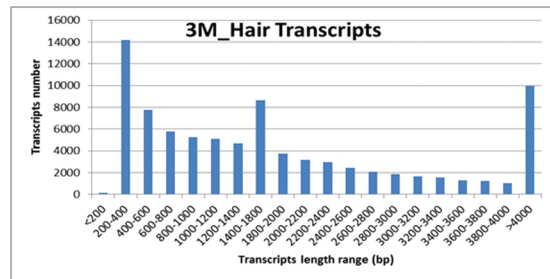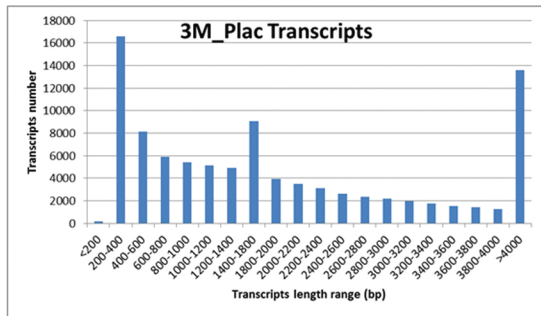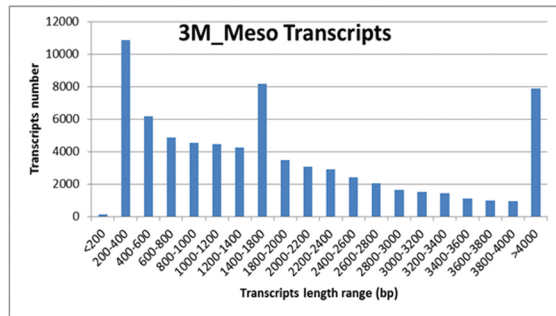

Supplement: Supplementary file 4 — Additional file 4: Length frequency distribution of the assembly transcripts from Seed, Hair, Meso and Plac in 3 month, respectively. (PDF 1 MB) [file 12864_2014_6656_MOESM4_ESM.pdf]

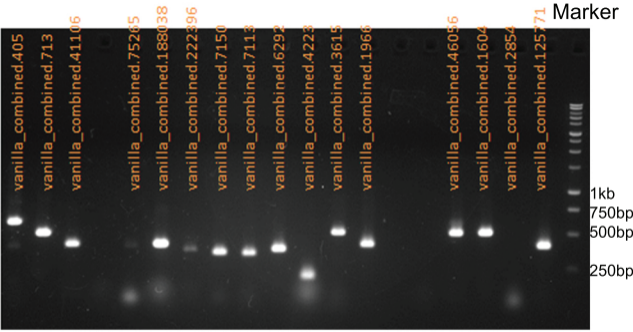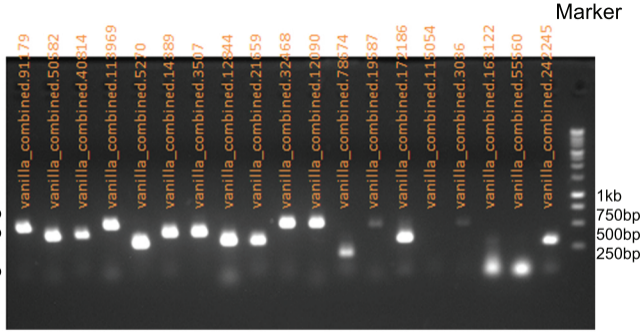

Supplement: Supplementary file 5 — Additional file 5: PCR validation for the hybrid-assembled transcripts. (PDF 1 MB) [file 12864_2014_6656_MOESM5_ESM.pdf]

A

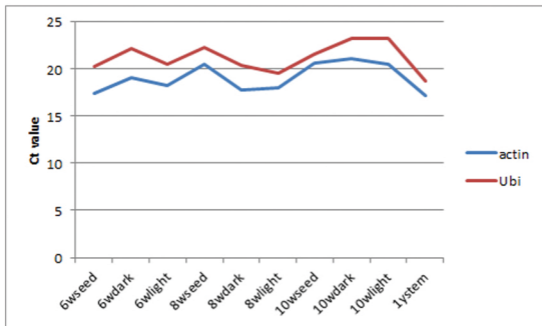

B

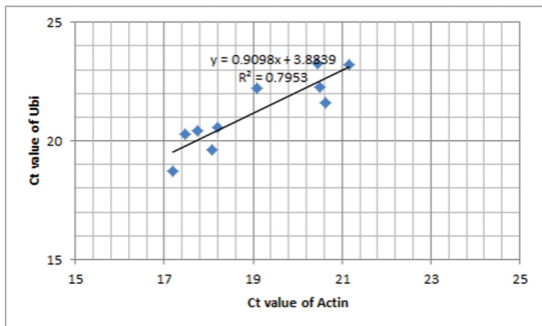

Supplement: Supplementary file 11 — Additional file 11: Q-PCR validation for Ubi and Actin. (A) Cq values of Ubi and Actin detected by Q-PCR in 6 weeks, 8 weeks, and 10 weeks of dark, light and seed tissues and in stems, (B) Correlation of Cq values of Ubi and Action. (PDF 843 KB) [file 12864_2014_6656_MOESM11_ESM.pdf]

## A. 6W seed

### biological\_process Level 3

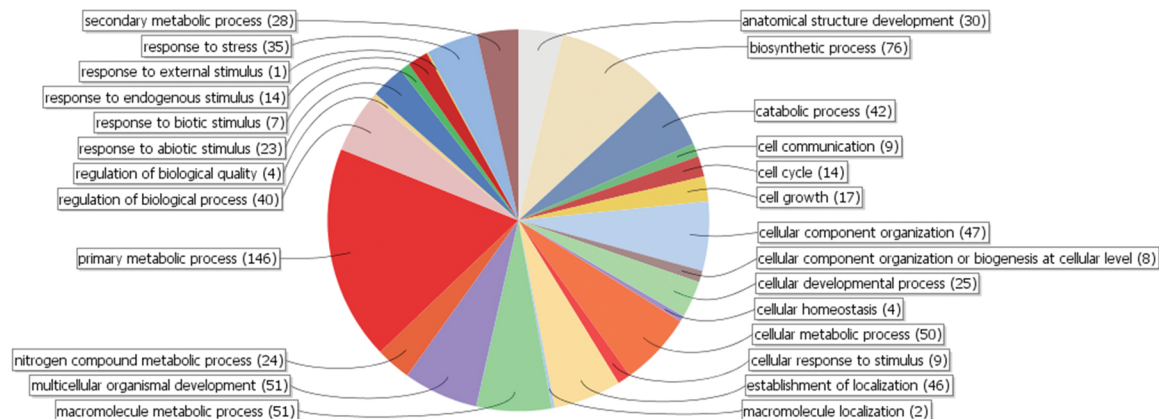

## B. Aerial root

### biological\_process Level 3

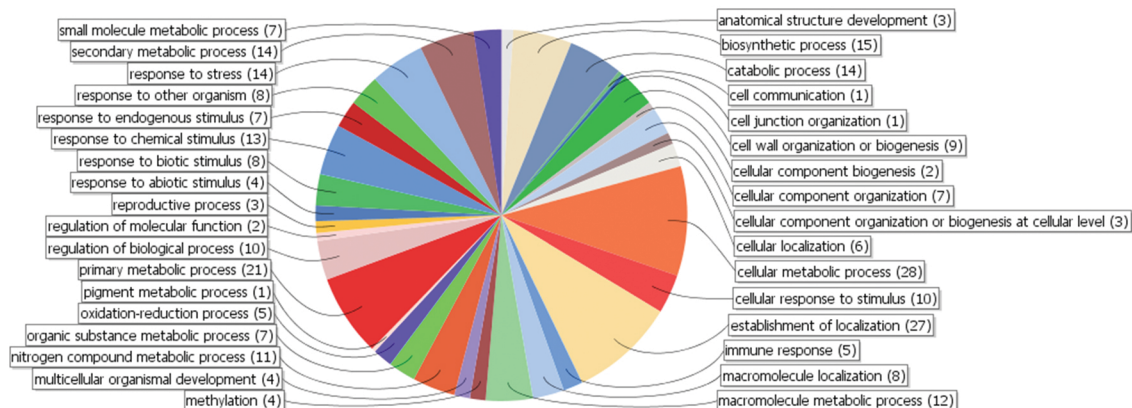

Supplement: Supplementary file 13 — Additional file 13: GO classification of tissue-specific genes in 6 week seeds and aerial roots. (PDF 2 MB) [file 12864_2014_6656_MOESM13_ESM.pdf]
